# The Hunt Lab Weighs in on Mass Spectrometry–Based Analysis of Protein Posttranslational Modifications

**DOI:** 10.1016/j.mcpro.2025.100943

**Published:** 2025-03-11

**Authors:** Joshua J. Coon, Jarrod A. Marto, John E.P. Syka, Forest M. White

**Affiliations:** 1 Department of Chemistry, University of Wisconsin-Madison, Madison, Wisconsin, USA; 2 Biomolecular Chemistry, University of Wisconsin-Madison, Madison, Wisconsin, USA; 3 Morgridge Institute for Research, Madison, Wisconsin, USA; 4 Department of Cancer Biology and the Linde Program in Cancer Chemical Biology, Dana-Farber Cancer Institute, Boston, Massachusetts, USA; 5 Blais Proteomics Center, Dana-Farber Cancer Institute, Boston, Massachusetts, USA; 6 Center for Emergent Drug Targets, Dana-Farber Cancer Institute, Boston, Massachusetts, USA; 7 Department of Pathology, Brigham and Women's Hospital and Harvard Medical School, Boston, Massachusetts, USA; 8 Thermo Fisher Scientific, San Jose, California, USA; 9 David H. Koch Institute for Integrative Cancer Research, Massachusetts Institute of Technology (MIT), Cambridge, Massachusetts, USA

**Keywords:** protein posttranslational modifications, phosphopeptide, electron transfer dissociation

## Abstract

Protein posttranslational modifications have traditionally been challenging to identify due to their dynamic regulation and typically low stoichiometry. Methods for phosphopeptide enrichment from complex proteomes developed in the Hunt lab in the late 1990's and early 2000's launched the field of phosphoproteomics, the large-scale analysis of protein phosphorylation sites. To improve phosphopeptide tandem mass spectra and address the further challenge of identifying other labile posttranslational modifications such as glycosylation or tyrosine sulfation, the Hunt lab invented and disseminated electron transfer dissociation, a novel method for peptide and protein fragmentation. Here we provide a brief historical accounting of these discoveries and their ensuing applications.

Protein post-translational modifications (PTMs) regulate almost all aspects of protein biology, including subcellular localization, protein folding and stability, protein–protein interactions, and enzymatic protein activity, among others. Hundreds of protein PTMs have been identified, ranging from relatively simple modifications such as arginine methylation to more complex structures such as lysine-bound poly-ADP ribosylation or asparagine-linked complex carbohydrates. Methodology developed in the Hunt lab in the late 1990's and early 2000's enabled the enrichment and facilitated identification of protein PTM's, leading to the large-scale systematic characterization of many of these modifications.

Among the legion of PTMs, the reversible, dynamic phosphorylation of serine, threonine, and tyrosine residues is perhaps the best studied (1, 2, 3). Indeed, the early recognition that protein phosphorylation played an important regulatory role and moreover that kinases were likely overrepresented as causal factors in several human cancers motivated an intense focus on the kinases (4). Molecular genetics and genomics has revealed much about kinases—starting with the first characterization of phosphorylase kinase in 1959 (5), the identification of the transforming potential of Src kinase in 1976 (6, 7, 8), and through the advances in cloning, sequencing, and bioinformatics in the 80s and 90s that led to the functional identification of many disease-associated kinases, ultimately culminating in additional information revealed by the publication of the human genome project in 2001 (9). However, validating the biochemical activity of individual kinases on specific Ser-/Thr-/Tyr-substrate residues *in vitro* remained a slow, typically one-substrate-at-a-time process (10, 11, 12). As we entered the postgenomic era, unbiased analysis of kinase activity within the endogenous cellular proteome was completely out of reach.

The challenge of identifying substrates and substrate phosphorylation sites for a given kinase was initially addressed by using radiolabeled [γ-^32^P]-ATP and recombinant kinases to assess the ability of a kinase to phosphorylate a given substrate in *in vitro* kinase assays. While these studies were able to identify kinase substrates, they failed to identify specific sites of phosphorylation without significant additional effort, and they required relatively large amounts of radiolabeled [γ -^32^P]-ATP and milligrams of recombinant protein substrate. The Hunt lab's development of peptide sequencing by low-energy collision-induced dissociation on a triple-quadrupole mass spectrometer (13) provided an alternative, in particular to the Edman-based sequencing of digested nonradiolabeled isoforms of the phosphorylated kinase substrate that were used to limit the spread of radiolabeled samples onto analytical instrumentation.

Mass spectrometry (MS)–based peptide sequencing approaches (14, 15, 16, 17) opened the door for the identification of phosphorylated peptides and phosphorylation sites with much higher throughput, from smaller amounts of sample (down to femtomole levels), and from mixtures of peptides and phosphopeptides—all without the need to purify the phosphopeptide for sequencing. However, they were still predominately limited to relatively simple mixtures such as proteolytic digests of single proteins. For much more complex mixtures, selective enrichment of phosphopeptides prior to MS analysis was needed to enable high throughput analysis of large numbers of phosphopeptides.

In the face of these challenges, in 1999, Donald Hunt (Don) asked Forest White, a postdoctoral associate in the Hunt lab, and Scott Ficarro, a graduate student in the Hunt lab, to develop an improved method for phosphopeptide enrichment. A few attempts with Fe^3+^-IMAC–based enrichment of phosphopeptides reproduced earlier findings with phosphopeptide enrichment working well in simple mixtures but plagued by nonspecific retention of nonphosphopeptides in more complex, biologically relevant mixtures (18, 19). During one of these analyses, Forest and Scott noted that most of the nonspecific retention was due to carboxylate-containing residues such as aspartic acid and glutamic acid, as most of the nonphosphorylated peptides had multiple acidic residues. Don suggested chemical modification of the carboxylate groups by conversion to methyl esters. Following this suggestion, Forest and Scott were able to virtually eliminate nonspecific binding of nonphosphopeptides to the Fe^3+^-IMAC column. In collaboration with Todd Stukenberg and Dan Burke, the Hunt group applied this new approach to *Saccharomyces cerevisiae* whole cell lysate, resulting in the identification of 383 phosphorylation sites in a single LC-MS/MS analysis (20). Despite the success of this project, some issues remained, including the dominant neutral loss of phosphoric acid from many of the phosphopeptide spectra. Experiments performed in the lab at the time by Scott and Forest highlighted the positive correlation between basicity of the peptide and neutral loss. As a result of this correlation, many of the sequenced peptides had multiple acidic residues and therefore provided additional peptide backbone fragmentation in the MS/MS spectrum.

Developments in Don's lab for selective phosphopeptide enrichment from complex proteomes provided a foundation and road map for continued technological innovation by his lab as well as other groups in two independent areas. First, as described further below, was the development of new electron-based dissociation techniques, originally motivated by the unique challenges of sequencing phosphorylated peptides by low-energy, collisionally activated dissociation. The second path focused on continued development and optimization of affinity chromatography techniques beyond the original IMAC platform to improve phosphopeptide yield and selectivity. Importantly, one critical area that was enabled by improved phosphopeptide enrichment was the identification of phosphorylated MHC peptide antigens, a field that was explored extensively by the Hunt lab, including experiments highlighting that phosphorylated peptides are immunogenic and may represent a form of neo-antigens (21, 22, 23, 24). In the ensuing years, the growing stable of phosphopeptide enrichment techniques were combined with diverse separation and MS platforms to rapidly quantify vast numbers of endogenous signaling events (3, 25, 26, 27). Collectively, these studies confirmed that as a functional compartment, the phosphoproteome was perhaps even more complex and more dynamic (28) than many had imagined. Due to these developments, we are well positioned to fill gaps in our understanding of kinase-substrate relationships and to define the mechanisms underlying the connection between kinase mutations and resulting alterations in cellular phenotypes. In fact, the overall impact of phosphoproteomic technologies is reflected in recent commentaries noting that our ability to identify and catalog new phosphorylation sites greatly outpaces our capacity to functionally annotate them (29, 30, 31).

As noted above, successfully enriching phosphorylated peptides only led to a new technical challenge: dissociating phosphorylated peptides in a sequence-informative way. The prevailing method at the time, ion trap collision-activated dissociation, often generated high levels of phosphoric acid neutral loss, resulting in spectra with minimal peptide backbone fragments needed to elucidate the peptide sequence (32). This challenge prompted strong interest in alternative dissociation methods and led to the series of events unfolded below across which the Hunt group invented and disseminated electron-transfer dissociation (ETD). Although the vast majority of sites listed in PhosphoSitePlus and other databases today have been generated by beam-type dissociation, ETD made its broadest impact on difficult-to-sequence PTMs.

To address this new technical challenge, Don took inspiration from electron-capture dissociation (ECD), first reported in the late nineties by Fred McLafferty's group (33). In the summer of 2001, Don challenged John Syka and Jarrod Marto to bring ECD to the ion trap. John, having considerable experience with making ions in the trap using electrons, was against attempting ECD because it would be extremely difficult to trap both peptide cations and thermal electrons at the same time. Instead, he proposed the use of anions as electron donors, since the trap can easily store similar *m/z* ions of opposite polarity.

This suggestion resonated with Don, whose lab in the 1970s had pioneered the technique of negative chemical ionization—work that won him the ASMS Distinguished Contribution Award in 1994. Given Don's strong background in ion chemistry and in particular electron capture by small molecules, he was agreeable to this approach. The idea was somewhat risky, as considerable literature by ion/ion reaction pioneer Scott McLuckey suggested this chemistry could not work (34, 35). That said, Jarrod, John, and Don believed that the reagent anions McLuckey had been able to test (which required injection through the ring electrode of 3D trap) were not ideal for the chemistry they desired.

Finding the right instrument to test this new hypothesis—the germ that would grow into ETD—posed another challenge. The linear ion trap FT-MS system that Jarrod and John had been building was not ideal nor were the lab's 3D ion traps, which would limit the range of usable anions (36). But the commercial linear ion trap from Thermo Fisher (37) was expected to arrive in 2003 and viewed as much more amenable system for modification, notwithstanding a few technical hurdles. Don and the team agreed to wait.

By the time the linear ion trap actually became available in fall 2003, Josh Coon had been recruited to the team. Upon his arrival in December 2002, Josh began working with John to design and fabricate the chemical ionization source that would be affixed to the backside of the commercial linear ion trap MS system due to arrive within a year. Meanwhile, John worked out the technology to allow for the simultaneous trapping of positive and negative ions along the axial dimension of the linear ion trap.

In this period, not everyone shared the team's enthusiasm for the potential of ETD. John got an earful of skepticism from a passerby at the factory in San Jose while he was testing the trapping method with his longtime colleague Jae Schwartz. Additionally, Don had included the linear ion trap ETD concept in his 2002 NIH R01 renewal application, which the study section did not elect to fund. Fortunately, the program officer found short-term bridge money to keep the lab moving, and once the team generated data, the revised proposal received a fundable score. All throughout this time, Don persisted and continued to provide resources and enthusiasm to the team for this work.

In fall 2003, a brand new, first-off-the-production-line quadrupole linear ion trap mass spectrometer (Finnigan LTQ) arrived at the Hunt lab—with a bang. John and Josh commenced to modify the instrument control code and add the new CI source, vacuum system, and control electronics. With reagent source mounted, the team attempted to bring the modified system under vacuum for the first time one October morning. Shortly after powering up the pumps, they heard a giant bang and felt glass raining down on us. Smoke billowed out of the added vacuum chamber where the 4-inch glass viewport had been. In his rush to hook up all the vacuum connections, Josh had swapped the inlet and outlet of an added mechanical pump so that the outlet of the pump was pressurizing the chamber. The entire system was covered with pump oil.

It was a costly 3 week delay. Don had to convince Thermo to replace the turbo pump and various parts on warranty! By early November, they had the system back together and attempted the first ion/ion reactions—having selected sulfur hexafluoride as the reagent anion. At first glance, it was a failure: only the well-described proton transfer chemistry was detected. But with careful averaging of the spectra, they found low-level evidence of ETD. A low-level contaminant, perhaps from residual pump oil, was likely driving the small amount of ETD. This success started a search for better reagents and within the next 5 months, they had identified multiple highly efficient ETD reagents (primarily polycyclic aromatic hydrocarbons) and published two manuscripts on this work (38, 39). This culmination of the project was presented at the 2004 ASMS conference in Nashville and featured results similar to what is currently practiced today with Figure 1 showing one of the actual slides from that ASMS presentation. A photo of Hunt and the ETD team posing with the initial ETD-enabled LTQ instrument is shown in Figure 2.

**Fig. 1:**
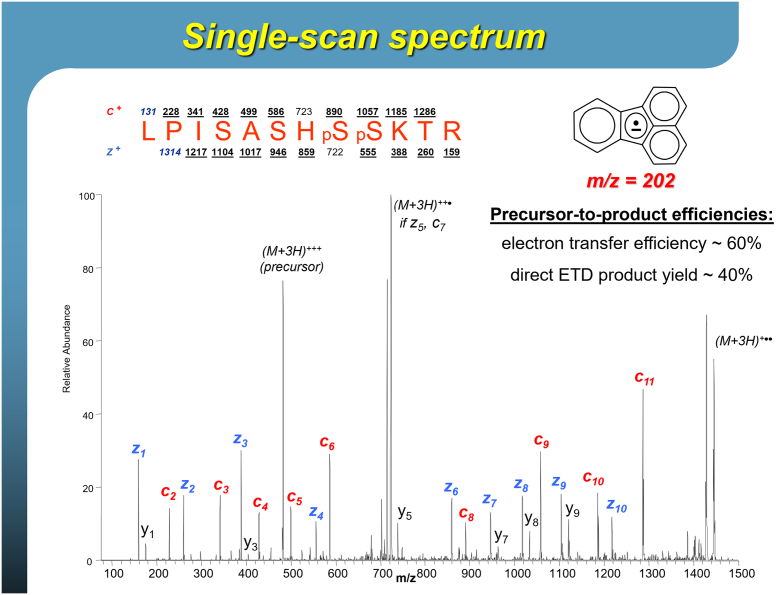
**Slide depicting an ETD MS/MS spectrum of a phosphopeptide thatwas presented at the 2004 ASMS Conference**.

**Fig. 2:**
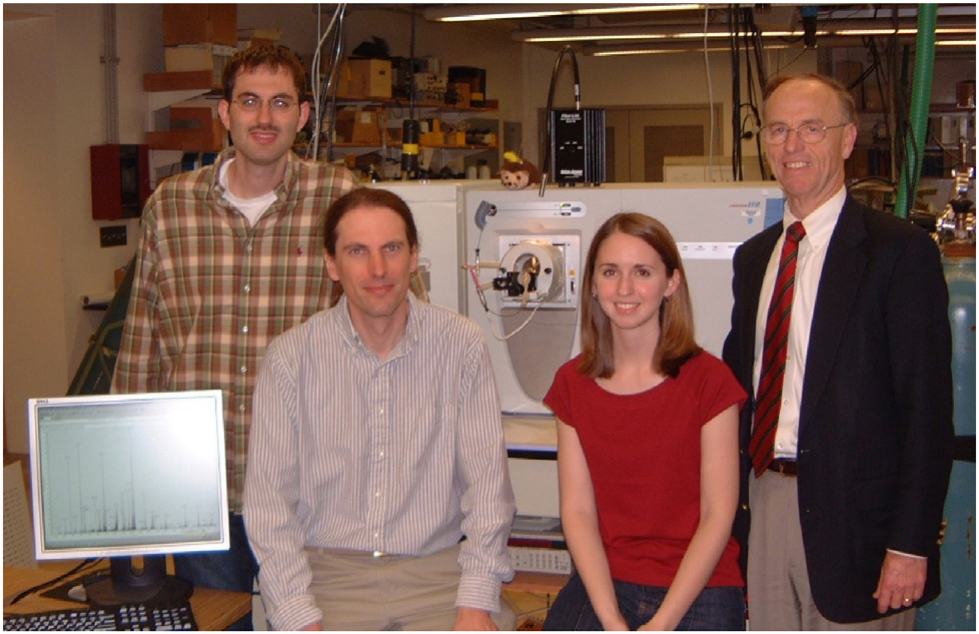
**Image of Josh Coon, John Syka, Melanie (Schroeder) Patterson, and Don Hunt from 2004 in front of ETD-enabled LTQ MS system.** An ETD spectrum is shown on the monitor.

Very quickly, the Hunt lab leveraged ETD for many challenging problems including phosphorylated peptides (40, 41), glycosylated peptides (42, 43, 44, 45, 46, 47, 48), modified histone tails (41, 49, 50, 51, 52, 53, 54, 55), and intact proteins (56, 57), among others. The coupling of ETD and proton transfer was also explored on the low-resolution ion trap systems, showing how intact protein analysis could now be directly tackled (49, 56, 58, 59). The field quickly expanded, ultimately making an especially notable impact in glycoproteomics (60, 61, 62, 63, 64, 65). In the ensuing years, the Hunt lab has continued to pioneer ion/ion chemistry and drive the development of ETD. Hunt's group, in collaboration with Thermo Fisher, developed the now commercial front-end implementation approach for anion generation (66, 67, 68). With well over 1000 instruments in the world today that can conduct ETD, this technology remains a key tool for many biomolecular sequencing applications ranging from antibody analysis (57, 69, 70, 71, 72, 73) to global glycosylation site mapping (44, 74, 75, 76) to proteome deep sequencing (77, 78).

The development of these foundational technologies reflects Don's notable characteristics as a scientist and leader. Don has a remarkable ability to identify important opportunities and to attract individuals to his laboratory to work on these problems and execute the vision. His perseverance in dealing with funding and scientific challenges throughout his career is exceptional, often running against the grain of prevailing wisdom and in favor of much more affordable, accessible instrumentation for tackling complex biological problems. Beyond ETD, Don has impacted just about every facet of biological MS as it is practiced today. His legacy is not only in the papers he published and people he trained, but in developing the concepts and approaches to protein MS that remain state of the art.

## Conflicts of interest

J. J. C. is a consultant for Thermo Fisher Scientific. All other authors declare no competing interests.
